# Self-awareness of depression and life events in three groups of patients: Psychotic depression, obsessive–compulsive disorder and chronic medical illness in North India

**DOI:** 10.4103/0019-5545.31558

**Published:** 2006

**Authors:** Anjali Gupta, Indu Bahadur, K.R. Gupta, Dinesh Bhugra

**Affiliations:** * Student, MA Psychology Student, University of Delhi, Delhi; ** Reader, Department of Psychology, Daulat Ram College, University of Delhi, Delhi; *** Consultant Psychiatrist, 44-A Brijpuri, Yamuna Nagar 135001, Haryana; **** Professor, Institute of Psychiatry, King's College, London

**Keywords:** Depression, obsessive–compulsive disorder, culture, North India

## Abstract

**Background:**

Depression is a common experience across cultures although not all languages have words describing depression.

**Aim:**

To identify patients' perception and awareness of depression as an illness.

**Methods:**

Sixty psychiatric patients (each with depression or obsessive–compulsive disorder [OCD]) were compared with 30 medical patients with chronic physical illness and assessed on levels of awareness of depression in relation to life events.

**Results:**

Life events were more in patients with OCD compared to other two groups. All the three groups of patients had major depression.

**Conclusion:**

Absence of help-seeking for depression in patients with OCD and physical illness possibly indicate low level of awareness of depression in these patients. The findings are discussed in context of clinical practice.

## INTRODUCTION

Life events are significant changes which have been associated with the onset of depression and occur in an individual's life producing stress and require a level of adaptability which influences coping strategies. Positive and negative life events contribute to stressors in a similar way and in vulnerable individuals may lead to the onset of depression. Measuring the impact and context of events has led to a link between life events and depression.^1^

Depression is a common disorder not only as a major disorder in its own right but also as a comorbid condition in a number of psychiatric and medical illnesses such as obsessive–compulsive disorder (OCD), chronic physical condition, etc. Depression is prevalent across cultures although symptoms of depression and their relative importance in different cultures vary.^2^,^3^ The prevalence of depression in various samples in India has varied from 1.6% to 3.8% and these depend on the source of sample, the diagnostic criteria and screening instruments used.

OCDs include obsessive thoughts, rituals, doubts and phobias and may have comorbid depression.^4^ In physical illness depression may occur due to the physical illness being a cause of psychiatric disorder or as a result of treatment. In a previous study from India using the same psychiatric clinic as the source for recruitment, the authors found that in 75 consecutive cases of depression family conflict, death and illness were the commonest life events.^2^ The relationship between life events and depression was studied in three different clinical conditions. The study aimed to identify patients' perception and awareness of the depression as an illness.

## METHOD

### Setting

The sample was collected from an urban clinical setting in North India. The clinic is one of three private clinics serving an industrial town with a population of over 500,000 with a similar number in the surrounding rural conurbations. The average number of cases seen daily is around 60, of which at least a quarter is new referrals. A majority of the patients seen are from low socioeconomic and educational status.

### Sample

A total of 60 psychiatric patients were recruited. These were consecutive attenders for depression and OCD. Of these, 30 each had diagnosis of psychotic depression or OCD based on the DSM-IV criteria.^5^ The diagnoses were made by KRG and assessments were conducted by AG who was blind to the diagnoses. Thirty medical patients with chronic physical illnesses such as hypertension, diabetes mellitus, and arthritis but with no diagnosed formal psychiatric disorder were recruited from a nearby GP clinic and matched broadly into the same age groups (±3 years).

### Assessment

Three instruments were used for assessing mental state and life events in addition to basic sociodemographic data:

1. Are you depressed checklist based on the Centre for Epidemiologic Studies Depression Scale. This is a self-reporting questionnaire used as screening instrument in community for assessing presence of major depressive disorder. High scores on this scale suggest moderate to high level of depression.
2. Hamilton Scale for Depression-21 (HDRS-21)^6^ was used to ascertain severity of depression.
3. Life Events Inventory.^7^ This is a checklist for assessing life events in a specific period generally over one year. This has previously been validated and used in India.^3^

The questionnaires AG used were in Hindi and questions were read to all the patients in view of their low literacy. Only patients over 17 years of age were approached with prior ethical approval.

### Statistical analysis

SPSS-X was used for analysis. Raw scores were compared using means and *t* test. Analysis of variance (ANOVA) was conducted to establish significance of difference.

## RESULTS

In each group 30 patients who presented consecutively were recruited. There were no refusers. Among the group who had depression, 23 (77%) were women and 7 (23%) were men. Among the OCD group, 19 (63%) were women and 11 (37%) were men and the gender ratio was equal in the group that had patients with chronic physical illness.

The mean age of the subjects across three groups was 43.5 years. The Mean score of depression across three groups on Are you depressed checklist was 48.83 for patients with psychotic depression, 38.27 for patients with OCD and 20.3 for patients with physical illness indicating presence of depression in all three groups with higher scores suggesting moderate to high level of depression. This when compared with HDRS scores, the mean scores on HDRS-21, were 30.20 (SD 6.06) for patients with psychotic depression, 22.83 (SD 7.81) for patients with OCD and 11.97 (SD 13.35) for patients with physical illnesses. Thus, depression as expected was most severe in psychotic depression patients but moderate to high level of depression was also noted in patients with OCD.

ANOVA indicated that between groups the F ratio was 49.77 (p<0.01). The gender differences were marked in that women were noted to be more depressed as compared to men. However, in terms of severity no gender difference could be seen particularly in patients with severe depression. The mean scores for women in depression was 30.17, in OCD it was 24.95 and in physical illness it was 13.13 compared with 30.28, 19.18 and 10.80 in men respectively (significant on *t* test for OCD at *t*=2.37, p<0.05).

Life events were rated from the previous year and the OCD group had higher average of life events at 20, with 14 in depression and 13 in physical illness. The more common life events were death or serious illness of a family member, and conflict within the family. In view of the small numbers these differences were not significant.

## DISCUSSION

There are a number of caveats which must be borne in mind prior to interpreting the results. Firstly, the numbers are small and are not entirely representative as they were collected from an outpatients setting. Secondly, the cases were not first onset, hence any findings must take this into account. Thirdly, this is cross-sectional data and any aetiological impact of life events in the aetiology cannot be ascertained. The diagnoses were made by the same clinician, which has its advantages and disadvantages. The heterogeneity of psychopathology within each diagnostic group makes generalizability problematic.

The negative life events seemed to have played role across all three groups with patients with OCD showing more life events. All the three groups showed presence of major depression as examined on the ‘Are you depressed checklist’ as well as HDRS. The higher scores on ‘Are you depressed checklist’ match with the severity scores on HDRS suggesting the higher responses on ‘Are you depressed checklist’ may be indicative of severity of depression. However, this needs to be studied systematically.

As expected the overall rate and severity of depression was much higher in psychotic depression patients but interestingly patients with OCD also observed to be having much higher rates of depression than reported in earlier studies.^4^,^8^,^9^ Since there was no premorbid data on prevalence of depression in this group, it is difficult to ascertain the extent of depression and to assess the primary or secondary status.

Sayar *et al.*^10^ had argued that depression is prevalent in patients with OCD but more prevalent among women. This may be another possible explanation in the present study. It is also observed that patients with physical illness had comparatively lower level of depression. This finding was similar to that in earlier studies.^11^,^12^ This being heterogenous group, the nature and severity of physical illness and its impact on depression cannot be clearly stated.

Despite the presence of depression in patients with OCD and physical illness, no treatment was sought for same in these groups of patients. This lack of help seeking possibly reflects the low level of awareness of depression in these groups. Lack of identification of symptoms of depression may have lead to lower level of awareness but this needs to be explored further. It is also possible that the symptoms of depression in patients with physical illness or in patients with OCD were attributed to primary pathology and not to depression. But since depression as a comorbidity in any group can significantly alter the morbidity as well as course of the illness; it is essential to look for and treat depression in these patients at every level of health care.

To conclude in spite of small sample size, some broad general points can be made. Life events played role across all three groups. All three groups showed depression of varying severity but help-seeking for depression was not seen in patients with OCD as well as those with physical illness which possibly indicate lower level of awareness of depression in these patients.
